# Theoretical Study of Vinyl-Sulfonate Monomers and Their Effect as the Dopants of Polyaniline Dimers

**DOI:** 10.3390/molecules27196353

**Published:** 2022-09-26

**Authors:** Isis Rodríguez-Sánchez, Alain S. Conejo-Dávila, Anayansi Estrada-Monje, Alejandro Vega-Rios, Erasto Armando Zaragoza-Contreras

**Affiliations:** 1 Unidad Profesional Interdisiplinaria de Ingeniería Campus Guanajuato del Instituto Politécnico Nacional, Mineral de Valenciana No. 200, Fraccionamiento Industrial Puerto Interior, Silao de la Victoria 36275, Mexico; 2 Departament of Engineering and Materials Chemistry, Centro de Investigación en Materiales Avanzados, S.C., Miguel de Cervantes No. 120, Complejo Industrial Chihuahua, Chihuahua 31136, Mexico; 3 Centro de Innovación Aplicada en Tecnologías Competitivas, Omega No. 201, Colonia Industrial Delta, León 37545, Mexico

**Keywords:** bifunctional monomer, vinyl-sulfonate monomer, density functional theory, polyaniline, doping study

## Abstract

Establishing the structure–property relationships of monomers and polymers via theoretical chemistry is vital for designing new polymer structures with a specific application. Developing bifunctional monomers with selective polymerizable sites is one of the strategies employed to obtain complex polymeric systems. In this work, a theoretical study on anilinium 2-acrylamide-2-methyl-1-propanesulfonate (ani-AMPS) and anilinium 4-styrenesulfonate (ani-SS) monomers and their respective doped polyaniline dimer (PAni-d AMPS or PAni-d SS) was performed. The study focused on understanding the susceptibility of the vinyl group to a radical attack and the conformation changes resulting from the coordinated covalent bond between sulfonate and aniliniun. Applying Density Functional Theory with the B3LYP functional and a basis set of 6 − 31 + G(d,p), the structures of the ani-AMPS, ani-SS, PAni-d AMPS, and PAni-d SS were optimized, and the different chemical descriptors were determined. The simulation showed that the reactivity of the vinyl group in the ani-AMPS is slightly higher. The sulfonate group undergoes a conformational change when bonding with PAni-d AMPS or PAni-d SS compared to its respective bifunctional monomer. Additionally, the electronegativity of PAni-d depends on the dopant’s structure. Thus, the bonded spacer between the vinyl and sulfonate groups (dopant) plays a notable role in the final characteristics of ani-AMPS, ani-SS, PAni-d AMPS, and PAni-d SS.

## 1. Introduction

Bifunctional monomers are precursors of polymers that contain at least two polymerizable sites. Commonly, these monomers are employed as crosslinking agents since both polymerizable sites react using the exact mechanism. Likewise, the development of bifunctional monomers with selective polymerization sites has been a strategy in the last decade to produce complex polymeric structures such as self-assembling graft copolymers [1] and surface grafted polymer brushes [2], or core-shell systems [3]. In these cases, a selective polymerization by one of the monomers occurs, while the other generates physical interactions between the polymer chains presenting new intrinsic properties different from conventional polymers.

Recently, we reported the synthesis of two bifunctional monomers, anilinium 2-acrylamide-2-methyl-1-propanesulfonate (ani-AMPS) [4] and anilinium 4-styrenesulfonate (ani-SS) [5]. In particular, these monomers contain two identical polymerizable sites (anilinium and vinyl); however, the spacers between the vinyl and sulfonate groups differ. Nevertheless, the polymers derived from these have different characteristics, such as molecular weight, glass transition temperature, conductivity, and UV-Vis absorption. However, these differences are impossible to comprehend with macromolecular chemistry; consequently, theoretical chemistry is a fundamental tool for such understanding.

Theoretical chemistry has been used to discern the monomer reactivity [6], polymerization mechanism [7], conformational arrangement [8], structure–property relationships [9], and others. In terms of the computational research of the anilinium group, examination of the π-stacking interaction and its crystalline structure [10], the development of ionic liquids [11], the oxidative polymerization mechanism [12], and the influence of the dopant on the polyaniline (PAni) properties has been achieved [13]. For example, Thirunavukkarasu et al. achieved a theoretical study of 2-acetyl-gamma-butyrolactone, reporting the most stable conformations of the monomers and dimers, intermolecular noncovalent interaction, and how these conformations and the interactions affect the UV-Vis absorption properties [14]. Likewise, Nifant’ev et al. simulated the copolymerization mechanism and conformations between maleic anhydride and α-olefines [15]. 

In this work, theoretical studies were carried out on ani-SS and ani-AMPS to determine the susceptibility of the vinyl group to a radical attack, the changes in the geometrical structure from bifunctional monomer to polyaniline dimers (PAni-d), and the dopant effect on a PAni-d. Furthermore, the most stable conformation of the two vinyl-sulfonate monomers is proposed, which was employed to determine their reactivity. Subsequently, the effects of these monomers as dopants of PAni-d were simulated. The study was complemented with the simulation of the FT-IR and UV-Vis spectra of the monomers and the systems of doped PAni-d.

## 2. Results and Discussion

The development of bifunctional monomers to produce complex polymeric systems has been a topic of our research group [4,5]. In particular, this research aims to analyze the reactivity of the alkene group to a radical attack and the effect of the sulfonate group as a doping agent of the polyaniline backbone. Applying the Density Functional Theory (DFT), with the B3LYP functional and a basis set of 6 − 31 + G(d,p), the most probable conformation of anilinium 4-styrenesulfonate (ani-SS) and anilinium 2-acrylamide-2-methyl-1-propanesulfonate (ani-AMPS) anions were analyzed. However, when an ionic molecule is constructed in Gaussian, the way a charge of +1 (N) is assigned is from the instruction in the calculation method. In addition, FT-IR and UV-Vis simulated spectra were obtained. 

### 2.1. Theoretical Structure of Monomers 

**Figure 1:**
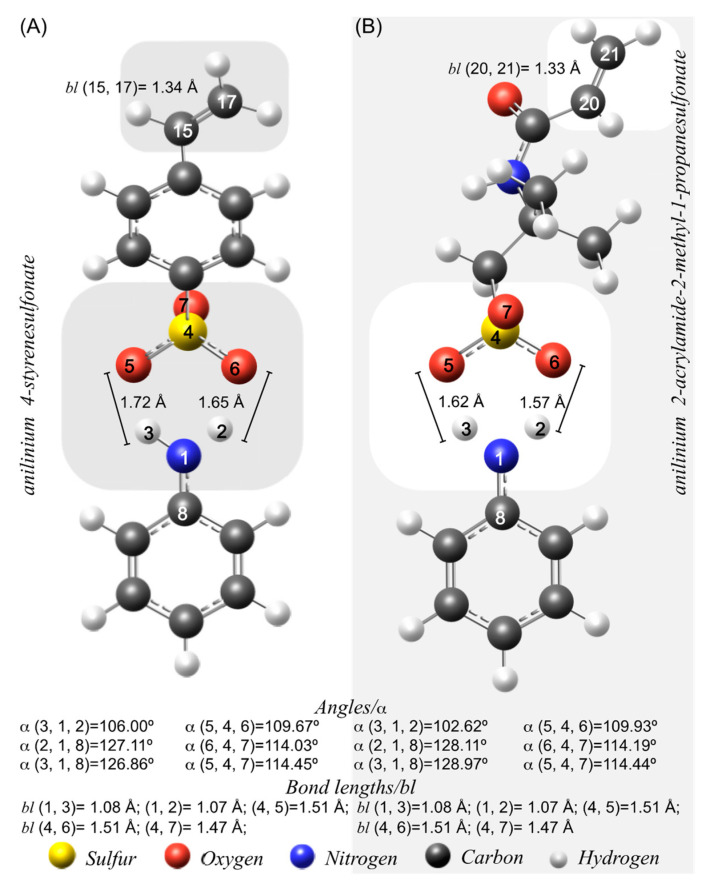
Bond angles and bond lengths of molecular models of (**A**) anilinium 4-styrenesulfonate (ani-SS) and (**B**) anilinium 2-acrylamide-2-methyl-1-propanesulfonate (ani-AMPS).

The molecular structure simulation describes the bond lengths and angles determined when the conformation of the molecules is at minimal energy. Figure 1 displays ani-SS and ani-AMPS bond lengths and angles, optimized at minimal energy. The vinyl bond and the sulfonate group were the subjects of interest in the analysis. Both monomers have similar bond lengths for the sulfonate group; however, AMPS presented slightly smaller bond angles. Equivalent studies can be consulted in the literature [16]. 

#### 2.1.1. Modeling of IR Spectra

**Figure 2:**
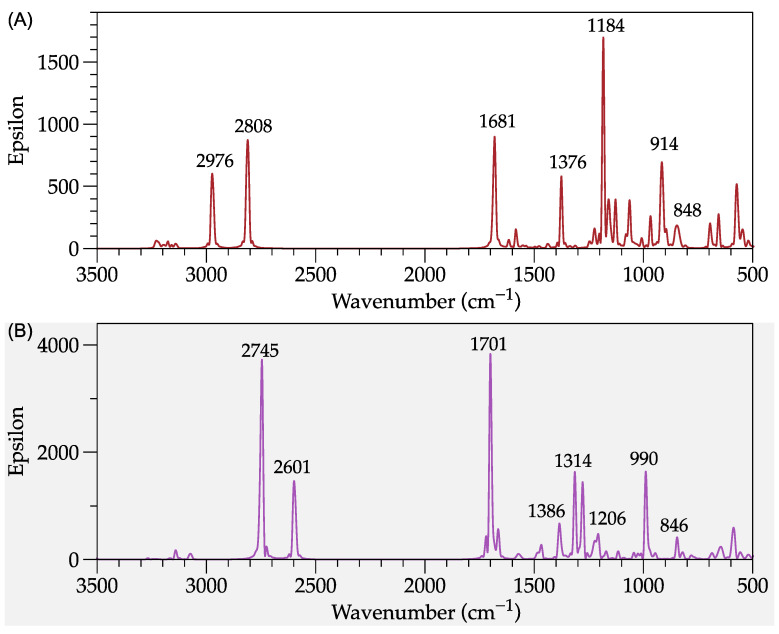
Modeled FT-IR spectra. (**A**) ani-SS; and (**B**) ani-AMPS.

Figure 2 compares the simulated infrared spectra of the ani-SS and ani-AMPS monomers, respectively. The study focused on finding the differences in the spectra from the analysis of the common groups of both structures, such as sulfonate, vinyl, and anilinium. The molecular vibrations of ani-SS corresponding to the asymmetric stretching of the anilinium group (N-H) are observed at 2808 and 2976 cm^−1^, while the ani-AMPS are at 2601 and 2745 cm^−1^. Likewise, the bands at 1681 and 1701 cm^−1^ are attributed to a bending vibration of N-H for the monomer ani-SS and ani-AMPS, respectively. This result can be explained because ani-SS presents a conjugated configuration with an electroattractive functional group (aromatic ring), while AMPS acts like a vinyl-amide. Regarding the vinyl group, it has been reported that vinyl groups show stretching absorption bands in the range of 1640–1645 cm^−1^ [17]. Both monomers have peaks in this region, specifically at 1681 and 1665 cm^−1^, for ani-SS and ani-AMPS, respectively. The simulation of the FT-IR spectrum of ani-SS also indicates an overlap of bands at 1681 cm^−1^. Similarly, ani-AMPS amide group vibration occurs at 1665 cm^−1^ [18].

Moreover, the sulfonate group of both monomers presents changes in intensity. The two typical bands of the sulfonate group are S-C and S=O stretching bands around ~1100 and ~920 cm^−1^, respectively [11]. In particular, for ani-SS, the peaks at 1184 and 1064 cm^−1^ are associated with the symmetrical and asymmetric stretching vibration of S=O, respectively. At 914 cm^−1^, the S-C stretching vibration of the sulfonate group is observed. In this region, peaks are associated with a bending vibration of the aromatic ring, suggesting that sulfonate interacts with the aromatic ring. Concerning the ani-AMPS monomer, the peaks at 1206 and 1170 cm^−1^ correspond to the symmetrical and asymmetrical vibration of the S=O, respectively. Meanwhile, the band at 990 cm^−1^ is associated with S-C (asymmetric stretching vibration).

On the other hand, the FT-IR spectrum of ani-SS presents, at low frequencies, between 675 and 900 cm^−1^, the out-of-plane vibration bending of the C-H bonds assigned to the aromatic ring. The in-plane deflections also appear around 1200 cm^−1^. In addition, the FT-IR spectrum of ani-SS shows the C=C stretching of the ring as doublet bands at 1585–1600 and 1400–1500 cm^−1^. Finally, the C-H vibrations ascribed to the aliphatic groups and the aromatic ring, for both monomers, occur between 900 and 1450 cm^−1^ and at 3000 cm^−1^ [19]. The simulated spectra of monomers, in general, are similar to those experimentally obtained [20,21].

#### 2.1.2. Global and Local Reactivity Descriptors

Qualitative chemical concepts such as electronegativity and hardness can be theoretically employed to examine various aspects of chemical reactivity, and a rigorous theoretical basis has been developed with DFT. These reactivity indices are notably appreciated in terms such as the principle of equalization of electronegativity, the acid–base principle and the principle of maximum hardness [22], and the principle of minimum polarizability, among others [23].

Electronegativity and hardness are two important descriptors of global reactivity used to understand the nature of the chemical bond. On the one hand, electronegativity is based on thermochemical data, exhibiting a direct correlation with the dipole moment, which measures the ionic character of a bond. According to Mulliken, it is commonly defined as absolute electronegativity since it does not depend on the molecular environment and can be obtained directly from two quantities that can be evaluated experimentally, that is, ionization potential and electron affinity of any atom or molecule [24].

Although global reactivity descriptors are widely utilized to describe the chemical behaviors of a system, it has been reported that local functions contain information about the inherent reactivity of molecules and specific control associated with a chemical reaction. The HSAB principle was originally formulated, according to DFT, for studying global changes within a reaction; nevertheless, it can also be applied to local interactions. The HSAB principle has been used to apprehend the selective sites in a molecule. This principle stipulates that a reaction site with high softness values might prefer to react with soft species or the softer site of a species. Conversely, a hard reaction site is expected to interact with hard–hard interactions. Soft–soft interactions are preferred in maximum Fukui functions, and minimal Fukui functions are chosen sites in hard–hard interactions [24].

Local reactivity descriptors have recently been used in the study of selective sites in a molecule. Because chemical reactions are dynamic processes, the time-dependent profiles of these descriptors and the dynamic counterpart of structural principles have been widely used to predict the behavior of a chemical reaction from start to finish. Fukui indices measure the sensitivity of a chemical potential generated within a system subjected to an external disturbance at a selective site. Fukui functions have discontinuous derivatives, so three different types of Fukui functions have been defined: nucleophilic, electrophilic, and radical attack [25].

Reactivity descriptors are qualitative and quantitative chemical concepts used to understand chemical reactivity. A rigorous DFT theoretical analysis has been developed using the local descriptors obtained from the condensed Fukui functions [24,26,27,28], according to the characteristics of the studied monomers. These reactivity indices are reported in terms such as the electronegativity equalization principle, the acid–base principle, the principle of maximum hardness, and the principle of minimum polarizability, among others [5]. 

As ani-SS and ani-AMPS perform as anions, they release energy by bonding to the PAni main chain. Table 1 illustrates the global electronegativity descriptor for the monomers. Furthermore, the global softness indicates how big the molecule is, which directly affects the molecule’s ability to be deformed. This is a direct relationship in which the higher the global softness value, the more sensitive it is to an external disturbance. In this case, the overall softness order indicates that SS is more significant than AMPS.

**Figure 3:**
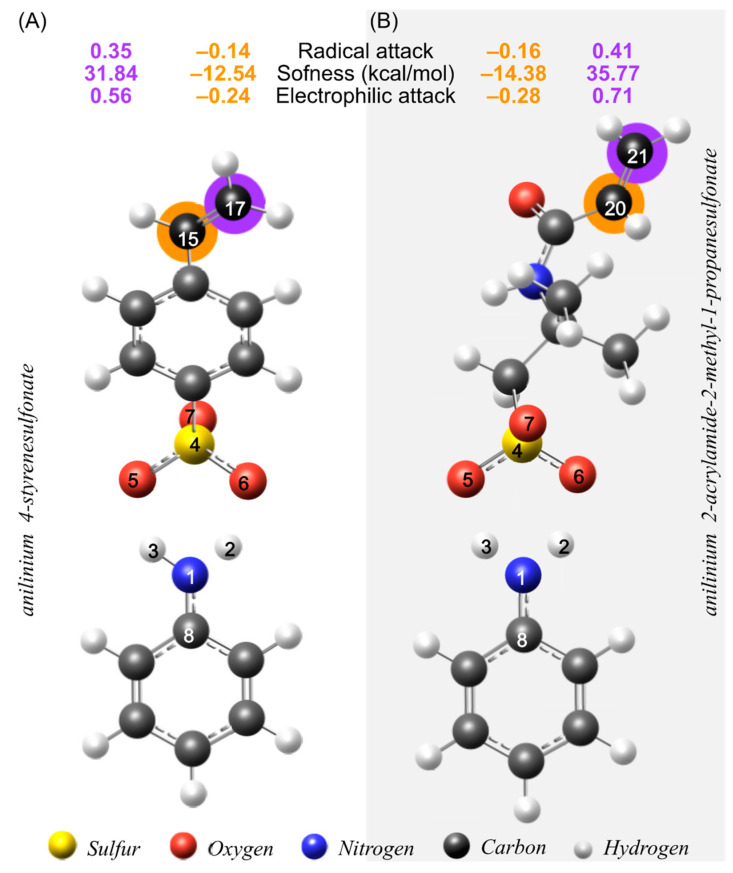
Reactive sites for free radical attacks. (**A**) ani-SS and (**B**) ani-AMPS.

The susceptibility of the double bond to an attack by free radicals is more remarkable for 17 C than 15 C, see Figure 3. Hence, 17 C is the most sensitive atom to an external energy disturbance related to a radical attack. On the contrary, AMPS has similar behavior to SS. The highest softness value corresponds to 21 C, which acts as a free-radical attack point.

Comparing the radical attack susceptibility of the SS (17 C) and AMPS (21 C), we can deduce that AMPS is more prone to reacting with a free radical. One possible explanation for this result is that the monomer SS (aromatic ring) spacer causes a decrease in its vinyl group’s electron density. This result coincides with the experimental results since the AMPS polymer has a higher conversion and molecular weight than the SS polymer [4,5]. 

### 2.2. Structural Simulation of Polyaniline Dimers with Dopants

**Figure 4:**
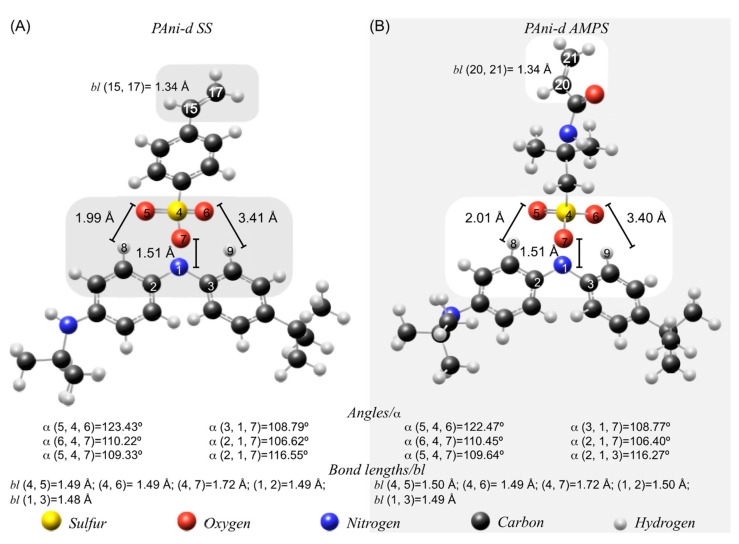
Geometric structure of (**A**) polyaniline dimer doped with styrene sulfonate (PAni-d SS) and (**B**) polyaniline dimer doped with 2-acrylamide-2-methyl-1-propanesulfonate (PAni-d AMPS).

Both anions have been dopants for the PAni [29,30]. As a consequence of the interaction of these anions (styrene sulfonate (SS) and 2-acrylamide-2-methyl-1-propanesulfonate (AMPS)) with the PAni backbone, a polyaniline dimer (PAni-d) with a dopant was simulated, and its structure was optimized [31,32,33]. Figure 4 illustrates the geometrical structure of the PAni-d doped with SS (PAni-d SS) and PAni-d doped with AMPS (PAni-d AMPS). In addition, the lengths and angles of the PAni-d SS and PAni-d AMPS emphasize the nitrogen and sulfonate bonds, which is the heteroatom that reacts with the SS and AMPS dopants. 

Moreover, the contrast of bond angles and lengths reveals that both structures have similar values. Nevertheless, a comparison with their respective monomers reveals that the distance between nitrogen (1) and the sulfonate group of the dopant decreases. Furthermore, PAni-d has interactions, for both structures, between two oxygens (5, 6) of the sulfonate functional group (SO_3_) and two hydrogens (8, 9) of the PAni-d backbone. The distance of this intermolecular force is 1.99 Å (H 8 to O 5) and 3.40 Å (H 9 to O 6).

Due to PAni polymerization, these changes can be attributed to a twisting of the C-S bond of the doping agents, until O 7 is aligned with N 1 causing an elongation of the sulfur-oxygen bond (7) and the increase in the angle α (5,4,6) compared to the monomers.

**Figure 5:**
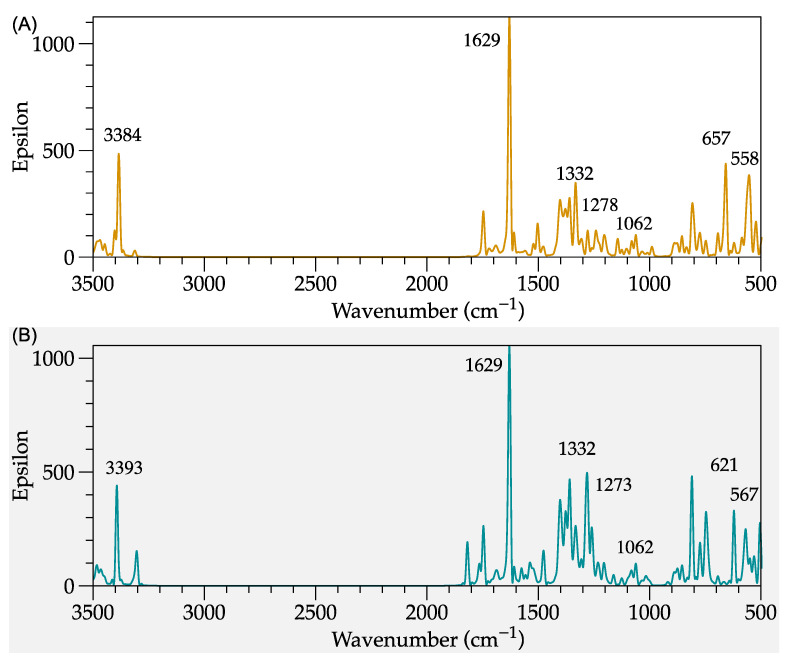
Theoretical infrared spectrum of (**A**) PAni-d SS and (**B**) PAni-d AMPS.

The computational study of PAni-d SS and PAni-d AMPS was complemented with the theoretical FT-IR spectrum, see Figure 5. The five signals corresponding to the vibrations of the C-N bond are observed around 3393, 1629, 1332, and 1273 cm^−1^ for PAni-d SS and PAni-d AMPS. The sulfate group also exhibits vibrations at typical frequencies, e.g., 1062, 621, and 567 cm^−1^. Compared to the theoretical spectra, the experimental ones including the bands corresponding to C-N are presented at 1230–1310 and 1503–1511 cm^−1^, and for C=N at 1579–1595 cm^−1^ [34]. 

#### Reactivity Calculation of PAni-d

The configuration of PAni-d presents an electron deficiency at the nitrogen atom, so it acts as a charge acceptor. On the other hand, it is also essential to grasp the interaction between anions and the PAni-d molecule and its ability to influence the capacity of this system with a band gap. The intention of these calculations is due to the need to analyze the behavior of the sulfonate group and its interactions with PAni-d, and to identify the structure that minimizes the conduction distance and promotes electronic exchange. Table 2 displays the global electronegativity descriptor for the PAni-d. 

The structural differences between monomers and doped PAni-d are reflected in global descriptors, mainly electronegativity. Compared with the ani-SS monomer, the PAni-d SS electronegativity increases; on the contrary, an opposite behavior occurs for the PAni-d AMPS and their monomer (ani-AMPS). This behavior is attributed to the difference in the type of spacer in each monomer, since the ani-SS is aromatic while the ani-AMPS is aliphatic. This behavior is also reflected in the philicity of the compounds. Similarly, the dimerization process of monomers is reflected in an increase in hardness and ionization potential, regardless of the doping agent presented in the dimer.

### 2.3. UV-Vis Spectrum

To simulate the UV-Vis spectra of PAni-d, PAni-d SS, and PAni-d AMPS with their respective monomers, an excited state energy calculation was made using the neutral state optimization.

**Figure 6:**
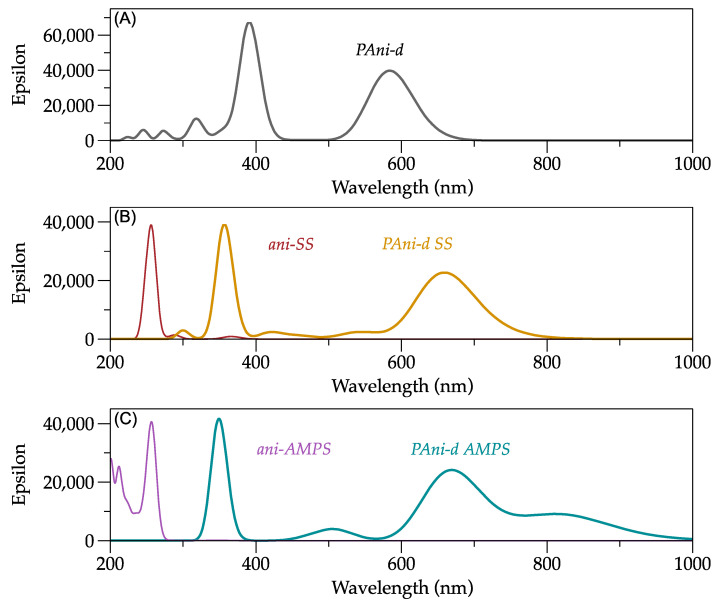
Theoretical UV-Vis spectra. (**A**) PAni-d; (**B**) ani-SS and PAni-d SS; (**C**) ani-AMPS and PAni-d AMPS.

The UV-Vis analysis exhibits the electronic transitions of the functional groups. For example, the PAni features three π-π* transitions, the first close to 348 nm, the second polaron-π* transition at 430 nm, and the third the π-polaron transition between 800 and 860 nm [35]. The PAni-d simulated presents three peaks at 424, 527, and 809 nm, similar to the experimental spectrum [36], see Figure 6A. 

Additionally, Figure 6B displays the simulated ani-SS spectrum and reveals a transition π-π* at 258 nm assigned to the aromatic rings (phenyl and anilinium). Compared to the ani-SS spectrum, the ani-AMPS spectrum highlights the band around 211 nm assigned to the n-π* carbonyl transition of the amide functional group.

Finally, Figure 6C shows the PAni-d AMPS and PAni-d SS spectra. A behavior similar, in general, is observed; nevertheless, the doped PAni-d presents the polaron-π* transition close to 355 nm, and the bipolaron-π* transition exhibits a bathochromic effect around 661 nm. 

#### Band Gap

The conduction capacity of a semiconductor depends significantly on the width of the gap or energy interval HOMO–LUMO [37]. The energetic barrier between HOMO and LUMO orbitals corresponds to donor and acceptor capacity. The band gap of PAni-d AMPS and PAni-d SS was calculated and compared with their respective monomers, see Table 3. The results show that the chemical structure of the dopant, specifically the spacer group, influences the band gap. In addition, the distance for PAni-d SS and PAni-d AMPS between O (7) of the sulfonate group (dopant) and N (1) of the PAni-d backbone is equal. Therefore, distance does not influence the band gap. Pousti et al. reported that an average approximation distance between silver and aniline is 2.37 Å using a computational and experimental study of polyaniline with the inclusion of silver atoms [38]. Similarly, Almasi et al. reported the elaboration of polyaniline/carbon nanotube nanocomposites using in situ chemical polymerization with a band gap of 2.84 eV utilizing 20 mg of carbon nanotubes [39]. Therefore, PAni-d SS presents a diminished band gap suggesting that the aromatic ring contributes to this phenomenon. 

## 3. Materials and Methods

The structures were theoretically optimized using the Density Functionals Theory (DFT) [40,41] and the functional PBE1PBE [42], with the set of bases 6 − 31 + G(d,p), achieving the obtention of the global minimum in the basal state gas phase on the surface of potential energy using Gaussian 09 [43]. The functional PBE1PBE [42], often called PBE0, presents a 25% Hartree Fock exchange not optimized empirically but instead based on perturbation theory arguments, so it is considered a functional free of adjusted parameters meaning that the results obtained are considered purely theoretical. With this functional, atomization energies, activation and reactivity enthalpies, ionization potentials, and electronic affinity are calculated, bond lengths were determined, among other parameters, and their efficiency was compared with some functionals used in DFT such as B3LYP [44,45]. After optimization and ensuring that no imaginary frequencies were presented, the geometric conformations were identified from the evaluated structures together with their IR and UV-Vis spectra. Finally, the analysis of global and local reactivity descriptors [24,26,27,28,46] was carried out to determine sites of chemical reactivity of interest. The visualization of the optimized structures, IR spectra, bond distances, and vibrations were carried out on the GaussViev^®^ 5.0.8 platform, and that of the UV-Vis spectra was carried out in SpecDis^®^ 1.71 [47].

Chemical reactivity studies involving global and local reactivity descriptors were performed to identify the reactivity and equilibrium constants. To achieve this study, two bifunctional monomeric radicals were evaluated in combination with a repeating polyaniline unit, only from the section where the polaron of the emeraldine salt is present, employing the DFT and the functional PBE1PBE [42], with the set of bases 6 − 31 + G(d,p). The study was also performed by combining the monomeric radicals with the polyaniline repeating unit.

## 4. Conclusions

In the present study, two monomers of anilinium vinyl sulfonate and polyaniline dimer (PAni-d) doped with their respective monomers were studied using theoretical chemistry. Firstly, the susceptibility of the alkene group to a radical attack was evaluated through an analysis of global reactivity descriptors. Furthermore, the anilinium 2-acrylamide-2-methyl-1-propanesulfonate (ani-AMPS) exhibited superior reactivity to a radical attack than the anilinium 4-styrenesulfonate (ani-SS) monomer. On the other hand, the changes in conformations of the dopant (sulfonate group) and PAni-d bond were analyzed through local reactivity descriptors. Specifically, the change in groups (sulfonate and amine) that interact in their monomeric form and PAni-d, highlighting the bond length and angles. The models exhibit a conformational change, when comparing PAni-d doped with their respective monomer. The spacer group located between the vinyl and sulfonate of the dopant plays a significant role in various parameters, such as a radical attack, electronegativity, hardness, philicity, and band gap. The models obtained from bifunctional monomers with anilinium allow us to propose complex systems from the interaction of polyaniline with different substrates. Future research will be focused in this direction with an impact on material chemistry.

## Figures and Tables

**Table 1:** Global descriptors of the ani-SS and ani-AMPS monomers, kcal/mol.

Monomers	Electron Affinity	Ionization Potential	Electronegativity	Hardness	Softness	Philicity
ani-SS	−64.47	94.71	15.12	79.59	0.00628	1.44
ani-AMPS	−63.33	101.34	19.00	82.33	0.00607	2.19

**Table 2:** Global descriptors of polyaniline dimers (PAni-d), kcal/mol.

Molecule	Electron Affinity	Ionization Potential	Electronegativity	Hardness	Softness	Philicity
PAni-d	−246.17	−129.33	−187.75	58.42	0.00856	301.71
PAni-d AMPS	−74.31	106.59	16.14	90.45	0.00553	1.44
PAni-d SS	−59.95	106.08	23.07	83.01	0.00602	3.20

**Table 3:** Band gap of ani-SS, ani-AMPS, PAni-d SS, and PAni-d AMPS.

Structure	Band Gap, eV
PAni-d SS	3.81
PAni-d AMPS	4.67
ani-SS	4.25
ani-AMPS	4.31

## Data Availability

Not applicable.
